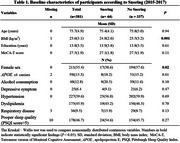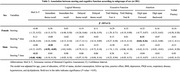# Association between snoring and cognitive impairment in community‐dwelling older adults in Taiwan

**DOI:** 10.1002/alz70860_099322

**Published:** 2025-12-23

**Authors:** Rui‐Qi Shi, Yen‐Ching Chen, Jeng‐Min Chiou, Jen‐Hau Chen

**Affiliations:** ^1^ Institution of Master Program of Statistics, National Taiwan University, Taipei, Taiwan; ^2^ National Taiwan University, Taipei, Taiwan; ^3^ Institute of Statistics and Data Science, National Taiwan University, Taipei, Taiwan; ^4^ Institute of Statistical Science, Academia Sinica, Taipei, Taiwan; ^5^ National Taiwan University Hospital Yunlin Branch, Yunlin, Taiwan

## Abstract

**Background:**

Previous studies have suggested that obstructive sleep apnea (OSA) may be related to cognitive function. However, the effect of snoring, as the most prominent characteristic of OSA, on cognitive function has not been fully explored. This study aimed to investigate the effect of snoring on cognitive function based on a seven‐year cohort study.

**Method:**

This cohort study (2015‐2022) using data from the Taiwan Initiatives for Geriatric Epidemiological Research collected at baseline (2015‐2017), involving 440 older adults aged 65 or older, with two follow‐up surveys conducted biennially. Cognitive function, including global cognition and specific domains (memory, attention, executive function, and language), was assessed at baseline and two follow‐ups using the Taiwanese version of the Montreal Cognitive Assessment and a battery of neuropsychological tests, respectively. The existence of snoring was obtained from the Pittsburgh Sleep Quality Index questionnaire, including both self‐reports and partner assessments at baseline. A generalized linear mixed model was employed to examine the association between snoring and cognitive performance, adjusting for age, sex, years of education, apolipoprotein E (*APOE) ε*4 status, depressive symptoms, alcohol consumption, practice effect, and follow‐up years. We further stratified the associations above by sex and age.

**Result:**

The mean age of this population is 75.7 at baseline and 55.4% were women. We found that body mass index, and sex significantly differed by the existence of snoring. Snoring was not associated with global or domain‐specific cognition. However, after stratification by sex, we found that, among men, baseline snoring was associated with the poor performance of global cognition over time (MoCA‐T, β = ‐0.33, 95% confidence interval=‐0.60, ‐0.05). In addition, snoring was associated with poor memory at baseline ( β = ‐0.50, 95% confidence interval=‐0.95, ‐0.05). In those aged >74 years, snoring‐time interaction was significantly linked to a decline in global cognition (MoCA‐T, β = ‐0.39, 95% CI = ‐0.70, ‐0.08).

**Conclusion:**

This study found that snoring had a significant detrimental effect on global cognition and memory in men but with an improvement in attention for women over time. Future research is warranted to investigate the sex differences and underlying mechanisms for the association of snoring with cognitive function.